# Indexing of Fatty Acids in Poultry Meat for Its Characterization in Healthy Human Nutrition: A Comprehensive Application of the Scientific Literature and New Proposals

**DOI:** 10.3390/nu14153110

**Published:** 2022-07-28

**Authors:** Alessandro Dal Bosco, Alice Cartoni Mancinelli, Gaetano Vaudo, Massimiliano Cavallo, Cesare Castellini, Simona Mattioli

**Affiliations:** 1 Department of Agricultural, Environmental and Food Science, University of Perugia, Borgo XX Giugno 74, 06124 Perugia, Italy; alessandro.dalbosco@unipg.it (A.D.B.); cesare.castellini@unipg.it (C.C.); simona.mattioli@unipg.it (S.M.); 2 Department of Medicine and Surgery, University of Perugia, Piazzale Gambuli 1, 06132 Perugia, Italy; gaetano.vaudo@unipg.it (G.V.); massimilianocavallotr@gmail.com (M.C.)

**Keywords:** fatty acids, chicken, meat quality, metabolism, human nutrition, index

## Abstract

Chicken meat is becoming the most consumed in the world for both economic and nutritional reasons; regarding the latter, the lipid profile may play positive or negative roles in the prevention and treatment of diseases. In this study, we define the state of the art of lipid-based nutritional indexes and used the lipid content and fatty acid profile (both qualitative and quantitative) of breast meat of two poultry genotypes with different growth rates and meat traits. Further, we summarize and review the definitions, implications, and applications of nutritional indexes used in recent years and others of our own design to provide a useful tool to researchers working in the field of meat quality (not only in poultry) to select the most appropriate index for their own scientific purposes. All indexes show advantages and disadvantages; hence, a rational choice should be applied to consider the nutritional effect of meat on human health and for a possible assessment of the most suitable rearing systems (genotype, feeding, farming system or postmortem handling).

## 1. Introduction

For many years, meat has been considered not only a source of nutritional elements such as proteins, lipids and minerals, but also a food capable of providing fundamental bioactive compounds for a whole series of metabolic functions that are at the basis of an optimal state of human health [1].

Many reviews have been published on the importance of the fatty acid composition of various meats and on the technologies used to improve their nutritional profile, mainly concerning the polyunsaturated fractions [2,3,4,5].

In these and other scientific studies, plentiful evidence has been given for all the metabolic functions that different fatty acids possess and in particular on the positive or negative roles in the prevention or onset of different chronic diseases, depending on their nature (i.e., presence or absence of double bonds and the number and/or position of them). Indeed, saturated fatty acids (SFAs) can increase the development of coronary heart diseases, multiple sclerosis and other metabolic dysfunctions [6], while polyunsaturated fatty acids (PUFAs) may have positive effects and beneficial actions on cardiovascular diseases, neurological diseases, allergic diseases, and so on [7].

These fatty acids are mainly obtained from various dietary sources, which have very different compositions and consequently have different effects on health outcomes. For this reason, the precise assessment of the fatty acid profile must be determined to rank foods based on their nutritional/functional properties, especially in fatty-acid-rich foods, food supplements and herb-based medicines.

It is well known that Long-Chain n-3 PUFAs (LC-PUFAs) are found predominantly in oily fish, but their consumption is declining [8], as well as that of all meats, with the exception of poultry, which has increased in consumption by 73% in the last 30 years [9]. From a nutritional point of view, comparing the n-3 PUFA content of poultry meat with that of mammal livestock, it seemingly has lower levels of n-3 and higher levels of n-6 [5], but it is also true that the fatty acid composition of poultry could easily be modified through feeding or management strategies [3,10,11]. In this scenario, organic or extensive rearing systems of poultry improve in the consideration of consumers, such as respect for animal welfare, environmental sustainability and food quality, due to the richness of some bioactive compounds, including n-3 PUFAs [9,12,13].

Based on the belief that the determination of the fatty acid profile, especially if expressed as a percentage, is not sufficient to explain the nutritional properties of a food and, taking a cue from a recent review on the nutritional indexes of meat and fish [14], we aimed to trace the state of the art of nutritional indexing, with indexes taken from the bibliography and other new ones of our conception. Therefore, the aim of the present study was to update the situation of lipid nutritional indexing in poultry meat and, on this basis, conceive other indexes that more effectively explore potential uses in the determination of nutritional properties by comparing two poultry genotypes with extremely different growth rates and fat contents. This study could help researchers working in the field of meat fatty acid composition to select the most appropriate index for their purposes. Indeed, after a reasonable evaluation of the best index, a more systematic research process has been used:

- to come to valid considerations about the nutritional effects of different meats on human health; and
- to provide a useful evaluation of the most suitable genotype and/or management practices (choice of the most appropriate feeding strategies, farming systems or post-mortem handling).

## 2. Materials and Methods

### 2.1. Animals and Diets

To obtain a complete dataset on lipid content and the profile to be indexed, an experimental trial was carried out at the poultry farm of the University of Perugia (Italy), using 100 subjects for each of two very different genotypes (50/50 sex ratio). In particular, the genotypes were extremely different in growth rate: slow-growing (SG, growth rhythm <20 g/d), originating from a conservation flock at the Department of Agricultural, Environmental and Food Science in the 1960s, and fast-growing (FG, growth rhythm >40 g/d), furnished by a commercial poultry farm (Avicola Berlanda, Italy). We chose these two divergent genotypes to differentiate the carcass and meat characteristics, particularly for lipid content and fatty acid composition (% of total fatty acids and mg/100 g) of meat (as reviewed by Dal Bosco et al. [15]), to verify the indexes considered in the present study.

All animals were reared according to EU regulation 834/07 on animal welfare for experimental and other scientific purposes. Furthermore, the experimental protocol was positively evaluated by an internal university committee (prot. 112606 of 12 January 2021). Chickens were kept separate after hatching until 20 d of age in an environmentally controlled poultry house with temperatures ranging from 20 to 32 °C and with relative humidity ranging from 65 to 75%. Incandescent light (30 lux) placed at the bird level was used for heating and illumination. Chicks were vaccinated against Marek and Newcastle diseases. At 21 days of age, the chicks were transferred to straw-bedded indoor pens (0.10 m^2^/bird), each equipped with feeders and drinkers and with free access to a forage paddock (4 m^2^/bird). Each genotype was represented in four replicates containing 25 chicks each. Birds were confined to indoor pens during the night. Chickens were fed ad libitum with the same starter (1–21 d) and grower–finisher (22 d to slaughter) diets (Table 1).

Access to feed and water was freely available, and all diets were formulated to contain adequate nutrient levels as defined by the NRC [16] and several authors [17]. Considering the very different growth rates of the birds, slaughter was carried out when 70% of the adult weight was reached, at approximately 110 days for SG and 45 days for FG.

### 2.2. Sampling and Analysis

A sample of 20 birds per strain, each weighing between ±10% of the population mean, was slaughtered in a slaughterhouse approved by the EU, 12 h after feed withdrawal. Chickens were electrically stunned (110 V; 350 Hz) before killing. After killing, the carcasses were placed in hot water (56.5 °C for 1 min) and then plucked, eviscerated (nonedible viscera: intestines, proventriculus, gall bladder, spleen, esophagus and full crop), and stored for 24 h at 4 °C. From the carcass, the pectoralis major muscle was excised for analysis. Samples were transported in refrigerated conditions to the department’s laboratory and immediately analyzed in duplicate to determine the lipid amounts. Total lipids were extracted in duplicate from 5 g of each homogenized sample and calculated gravimetrically [18]. Fatty acids were quantified as methyl esters (FAMEs) with a gas chromatography system (CP 3800 VARIAN, Varian Medical Systems Italia S.P.A., Milan, Italy) equipped with an FID detector and a capillary column of 100 m length × 0.25 mm × 0.2 μm film (Supelco, Bellefonte, PA, USA). To calculate the amount of each FA, heneicosanoic acid was used as the internal standard (C21:0, Sigma—Aldrich analytical standard, Steinfeld, Germany), and data were expressed as mg/100 g of meat (quantitative evaluation) and % of total FA (qualitative evaluation). The average amount of each fatty acid was used to calculate the sum of the SFAs, monounsaturated fatty acids (MUFAs) and PUFAs.

### 2.3. Indexes

All the indexes considered in this review were categorized into subgroups based on the aim for which they were conceived, and in particular into qualitative, nutritional, metabolic and lipid- or energy-related content indexes, as summarized in Table 2.

#### 2.3.1. Qualitative Indexes

##### Polyunsaturated Fatty Acid/Saturated Fatty Acid (PUFA/SFA)

PUFA/SFA = (Σ Polyunsaturated Fatty Acids)/(Σ Saturated Fatty Acids)(1)

The index reported in (1) is the most-used index for evaluating the impact of a particular food on cardiovascular health, assuming that all PUFAs are able to reduce low-density lipoprotein cholesterol and serum cholesterol, whereas all SFAs can contribute to increasing serum cholesterol. Thus, this is a direct index: higher values indicate a better (positive) effect (or the contrary), given by a certain meat or meat product intake.

##### n-6/n-3 Ratio

n-6/n-3 = (C18:2n-6 + C20:2n-6 + C20:3n-6 + C20:4n-6)/ (C18:3n-3 + C20:3n-3 + C20:5n-3 + C22:5n-3 + C22:6n3)(2)

As a complement to the aforementioned index, it could be coupled with the n-6/n-3 ratio (2) defined by Simopoulus [1,22], which has now become a way of evaluating the nutritional quality of foods used by thousands of researchers. A lower ratio of n-6/n-3 fatty acids is more desirable for reducing the risk of many of the chronic diseases of high prevalence in Western societies, as well as in developing countries [23].

##### Linoleic Acid/α-Linolenic Acid (LA/ALA) Ratio

LA/ALA = (C18:2n-6)/(C18:3n-3)(3)

This ratio (3) was developed as an evaluation tool for dietary infant formula. These two fatty acids cannot be synthesized in the mammalian body hence, they must be obtained by diet. Moreover, they compete for the same desaturase and elongase enzymes, which permit the synthesis of LC-PUFAs. Due to the low conversion rate of ALA, the LA/ALA ratio reduction only provides a modest improvement in the levels of some n-3 LC-PUFAs (i.e., EPA, DPA and DHA; [2]); thus, the index can be considered a first step for LC-PUFA estimation.

##### Sum of Eicosapentaenoic Acid and Docosahexaenoic Acid (EPA + DHA%)

EPA + DHA% = %C20:5n-3 + %C22:6n-3(4)

The two fatty acids of this index (4) are n-3 LC-PUFAs involved and positively correlated with human health, mainly in the reduction of the risk of cardiovascular diseases, hypertension, inflammation [3] and reproductive functions [24,25].

##### Unsaturation Index (UI)

UI = (% monoenoic) + (2 X % dienoic) + (3 X % trienoic) + (4 X % tetraenoic) + (5 X % pentaenoic) + (6 X % hesaenoic)(5)

This index (5) adds information on the degree of unsaturation of fatty acids rather than dwelling only on the sum of them. Indeed, UI indicates the degree of unsaturation of each fatty acid and is calculated as the sum of each unsaturated fatty acid (%) multiplied by the number of its double bonds, thus giving different weights to the different unsaturated classes. This index does not distinguish the two different series of fatty acids (n-3 and n-6); hence, it is not very specific for nutritional aspects, while it is of great importance for establishing the oxidative stability of livestock feed or human food and defining some oxidative protection strategies [5].

#### 2.3.2. Nutritional Indexes

##### Nutrition Value Index

Nutrition Value Index = (C18:0 + C18:1n9)/(C16:0)(6)

This index (6) was developed by Chen and coworkers [6]; it considers only the dominant fatty acids in a food of animal origin: palmitic (C16:0), stearic (C18:0) and oleic (C18:1n-9) acids, due to their higher concentrations.

##### Index of Atherogenicity (IA)

IA = [C12:0 + (4 × C14:0) + C16:0]/(Σ UFA)(7)

This index (7) was developed by Ulbricht and Southgate in 1991 [7], who published an article in *The Lancet* aimed to characterize the atherogenic potential of fatty acids of foods. The two researchers wanted to define a more specific index compared to PUFA/SFA, considered too general and weak an indicator to assess the atherogenicity of food. In particular, the IA indicates the relationship between the sum of SFAs, with the exception of stearic acid (C18:0), not considered pro-atherogenic by the authors because of the capacity of humans to desaturate it to oleic acid (C18:1n-9). In contrast, lauric (C12:0), myristic (C14:0) and palmitic (C16:0) acids favor the adhesion of lipids to cells of the circulatory and immunological systems and the accumulation of atherogenic plaques, and they reduce the levels of phospholipids and esterified fatty acids [26]. The IA has been used widely for evaluating seaweeds, crops, meat, fish and dairy products. Contrary to the previous index, a lower value indicates better nutritional characteristics of the food.

##### Index of Thrombogenicity (IT)

IT = (C14:0 + C16:0 + C18:0)/[(0.5 × MUFA) + (0.5 × Σn-6 PUFA) + (3 × Σn-3 PUFA) + (Σn-3 PUFA/Σn-6 PUFA)](8)

This index (8) was also developed by Ulbricht and Southgate [7], together with IA, to further characterize the thrombogenic potential of fatty acids, separating them on the basis of the effects triggered by some derivatives (eicosanoids) in pro-thrombogenic (C12:0, C14:0, and C16:0) and anti-thrombogenic FAs, such as MUFAs, n-3 and n-6 PUFAs, although current studies have demonstrated the negative implication of n-6 PUFAs on thrombogenesis [27]. Therefore, the consumption of foods or products with a lower IT is beneficial for human health (indirect index).

##### Hypocholesterolemic/Hypercholesterolemic (HH) Ratio

HH ratio = (C18:1 + Σ PUFA)/(C12:0 + C14:0 + C16:0)(9)

In 2002, Santos-Silva et al. [8] proposed this index (9) following their studies on lamb meat to better assess the nutritional properties of this meat. The authors developed the HH with the intention of focusing on the relationships between dietary fatty acids and plasma low-density lipoproteins relating the hypocholesterolemic fatty acids (C18:1n-9 and PUFA) and hypercholesterolemic fatty acids (C12:0, C14:0, C16:0).

##### Health-Promoting Index (HPI)

HPI = (Σ UFA)/[C12:0 + (4 × C14:0) + C16:0](10)

In 2004, Chen and coworkers [9] proposed this index (10) to assess the nutritional value of fat, with particular emphasis on the effects of fatty acids on cardiovascular diseases. It is immediately evident that this index is exactly the inverse of the IA and thus has become a direct index mainly used in research on dairy products.

##### Fish Lipid Quality/Flesh Lipid Quality (FLQ)

FLQ = 100 × (C20:5n-3 + C22:6n-3)/(Σ Saturated Fatty Acids)(11)

This index (11) was originally used for assessing the lipid quality of fish or flesh [28,29]. Its aim is similar to that of the sum EPA + DHA, but the sum of EPA and DHA is calculated as a percentage of fatty acids. It can be used as an ancillary to EPA + DHA since the absolute quantity for EPA and DHA is more important.

#### 2.3.3. Metabolic Indexes

Several indexes were used to estimate the activities of desaturases and elongases of muscle tissue through the enzyme approach “products/substrate ratio” [30].

The elongase index (12) was calculated as the ratio of C18:0 to C16:0, whereas the thioesterase index (13) was calculated as the ratio of C16:0 to myristic acid (C14:0) [12].

Elongase = ((C18:0)/(C16:0)) × 100(12)

Thioesterase = ((C16:0)/(C14:0)) × 100(13)

Estimated desaturase activities (14–15) are often used, and among many authors, Vessby et al. [13] reported that the calculated activities of Δ9-, Δ5- and Δ6-desaturase can be used as surrogates of the measure of true desaturase activity in the laboratory [31].

Δ9-desaturase (C:18:1) = ((C18:1n-9)/(C18:0 + C18:1n-9)) × 100(14)

Δ9-desaturase (C16:1 + C:18:1) = ((C16:1n-7 + C18:1n-9)/(C16:0 + C18:0 +  C16:1n-7 + C18:1n-9)) × 100(15)

To evaluate the activities of both Δ5- and Δ6-desaturase (16), the enzymes catalyzing the formation of long-chain n-6 and n-3 polyunsaturated fatty acids (PUFAs) starting from the precursors C18:2n-6 and C18:3n-3, the following equation was used [14]:

Δ5-desaturase + Δ6-desaturase = (C20:2n-6 + C20:4n-6 + C20:5n-3 + C22:5n-3 + C22:6n-3)/(C18:2n-6 + C18:3n-3+ C20:2n-6 + C20:4n-6 + C20:5n-3 + C22:5n-3 + C22:6n-3)(16)

In this view, the rate of n-3 β-oxidation in the muscle (e.g., n-3 LC-PUFA/ALA) can adequately describe n-3 LC-PUFA mobilization used for energy production (i.e., movement; [32]) and the resulting oxidative status. The ratio between n-3 LC-PUFA and ALA could be taken as an indicator of energy consumption (β-oxidation; (17)). This should be higher in oxidative than in glycolytic muscles [19] and therefore in animals with higher kinetic activity [33].

Activity Index = (Σ n-3 PUFA)/(C18:3n-3)(17)

#### 2.3.4. Lipid- or Energy-Related Content Indexes

##### Sum of Eicosapentaenoic Acid and Docosahexaenoic Acid (EPA + DHA Quantity)

EPA + DHA quantity = mg/100 g C20:5n-3 + mg/100 g C22:6n-3(18)

In 1994, Godbe [20] developed the first (18) of these last groups of indexes, while the other three have been conceived by us. These indexes differ from the others because they consider the quantity of fat or energy that is related to the quantity/quality of fatty acids in the food. We also calculated the percentage with respect to the daily requirements of various lipid classes, especially PUFAs, in a 150 g standard portion of chicken meat. This standard meal contains approximately 30 g of proteins, which is 35% of the 1800 kcal diet, based on 55:35:20% contents of carbohydrates, lipids and proteins [34]. To better understand the nutritional value of chicken meat lipid content in a real-life setting, we propose to examine the implication (in %) with respect to an ideal healthy meal in the context of a balanced Mediterranean Diet.

##### Index of Nutritional Quality

The index of nutritional quality (INQ; (19)) was calculated based on the eicosapentaenoic (EPA) + docosahexaenoic (DHA) acid content, considering the energy content of the meat (expressed as Kcal of every nutrient) and the EPA and DHA daily requirements.

INQ = (((Σ mg/100 g EPA + DHA))/(Energy content Kcal 100 g))/(EPA + DHA daily requirement)(19)

##### Proposed New Indexes

In the following indexes, our intention was to relate the fatty-acid profile with the total lipid content, in the belief that these nutritional properties cannot be evaluated separately. We propose a deeper lipid study through the development of three progressive indexes.

##### QuantiN-3 Index

In particular, the quantiN-3 index (20) relates the polyunsaturated fatty acids of the n-3 series expressed in quantity (mg/100 g) of meat with the quantity (g/100 g) of fat.

QuantiN-3 index = (mg/100 g of n-3 PUFA)/(g/100 g of Total Lipids)(20)

##### Healthy Fatty Indexes 1 and 2

In the two latter indexes, we wanted to further deepen the previous quantiN-3 index through the careful differentiation of the various classes of fatty acids (by unsaturation and by the position of the double bonds), partly following the indications of Ulbricht and Southgate [7] but always relating everything to the quantity of lipids (Healthy Fatty Index 1—HFI1, (21)). Furthermore, in Healthy Fatty Index 2 (22), the contents of the different classes of fatty acids and their properties were considered. The rationale of this last index is to consider the dietary lipid input not only from a nutritional viewpoint but also with a health approach. In particular, the idea is to consider the various classes of fatty acids by increasing or decreasing (using some empirically unknown constants: MUFA = 2; n-6 PUFA = 4; n-3 PUFA = 8, partly derived from Ulbricht and Southgate [7]) their relative content expressed in weight according to their health impact, deduced from the consolidated scientific literature.

The resulting value is divided by the fat, weighted on the quantity of various classes (SFAs, MUFAs, n-3 PUFAs and n-6 PUFAs). This decreases the values because of their negative effects on human health through specific constants (reciprocal of the constants in the numerator, i.e., SFA = 1, MUFA = 1/2 = 0.5, n-6 PUFA = 1/4 = 0.25, n-3 PUFA = 1/8 = 0.125). From this perspective, more weight is given to the n-3/n-6 ratio (or reversed): specifically, the numerator reports variables that accentuate the positive value of n-3 fatty acids (i.e., MUFA, n-6 PUFA, n-3 PUFA and n-3/n-6), and in the denominator, we amplify the lower health effect by introducing the weight of fatty acid classes. In particular, we enhance the n-6 PUFA effects by reversing the ratio (n-6/n-3).

HFI1 = ((mg/100 g of MUFA × 2) + (mg/100 g of n-6 × 4) + (mg/100 g of n-3 × 8) + (mg/100 g n-3/mg/100 g n-6))/(mg/100 g of Total Lipids)(21)

HFI2 = ((mg/100 g of MUFA × 2) + (mg/100 g of n-6 × 4) + (mg/100 g of n-3 × 8) + (mg/100 g n-3/mg/100 g n-6))/((mg/100 g of SFA) + (mg/100 g of MUFA × 0.5) + (mg/100 g of n-6 × 0.25) + (mg/100 g of n-3 × 0.125) + (mg/100 g n-6/mg/100 g n-3))(22)

### 2.4. Statistical Analyses

The data were analyzed with a linear model (STATA, 2015, College Station, TX, USA; [35]) to evaluate the effect of strain; the significance of differences (*p* < 0.05; *p* < 0.0001) was evaluated by Bonferroni multiple *t*-tests.

## 3. Results and Discussion

### 3.1. Fatty Acid Composition (The Dataset)

The lipid contents and the fatty acid profiles of the breast meat are shown in Table 3, expressed as % or in quantitative form (mg/100 g f.m.). The analysis of these results shows that the initial objective of the experiment was largely achieved; indeed, the choice of two divergent genotypes resulted in different lipid contents and fatty acid profiles.

As reported in our review [15], the genotype interacts with movement, intake of antioxidants, antioxidant capacity of the body, plasma, and fatty acid profile of meat. In particular, the SG chickens had a high kinetic behavior that may be matched with a more exploratory attitude, improving their meat’s nutritional characteristics. In contrast, FG chickens showed some kinetic problems, especially in the last phase of the cycle. The strain effect reported herein also implicates a different behavior of birds in terms of the amount of feed/forage intake. These genotypes, which were selected for high precocity and ability to reach high live weight at an early age, are not compatible with longer periods of raising [36]. The combination of age, low kinetic activity and high feed intake resulted in higher fat accumulation in muscles.

Breeds were also a source of variation for the main fatty acids. Differences in the fatty acid profiles were observed in the content of SFAs, where a higher value was observed in SG chickens and a lower value was observed in commercial lines. Additionally, SG chickens exhibited higher amounts of stearic acid (C18:0). In general, these strains produce very lean meats; despite the anatomical cut (breast) and the absence of skin, the difference between the genotypes was very significant (*p* < 0.0001), with an obvious modification of fatty acid amount. As an example, the sum of the n-3 PUFAs, which was double in the breast meat of SG subjects, was three times higher in the FG subjects from a quantitative point of view.

The MUFAs, which in chickens are related either to endogenous synthesis or to gut absorption from the diet, showed the highest levels in FG genotypes. These MUFAs were mainly represented by oleic and palmitoleic acids. The low MUFA level observed in SG genotypes can be attributed to the higher intake of pasture (average MUFA value 227 mg/100 g) with respect to the feed (average MUFA value 1134 mg/100 g) and to the different intramuscular fat content of birds [14]. Meat of slow-growing genotypes compared with fast-growing ones was characterized by a high percentage of PUFAs (both n-3 and n-6). Despite the increased consumption of fresh forage, lower levels of ALA in the meat of slow-growing strains could be explained by the higher conversion of this fatty acid in the long-chain derivatives [37].

### 3.2. Indexes

The different indexes are shown in Table 4 and are discussed following complexity and dealing with general and specific considerations for the meat of the two chicken genotypes. As already stated, all indexes were grouped into subcategories based on their underlying qualitative, nutritional and metabolic principles and considering the relationships between fatty acids and lipid- or energy content-related indexes of meat. The large difference in values obtained with the different indexes strongly underlines their different meanings and thus the use of each index in different conditions.

### 3.3. Qualitative Indexes

Concerning the PUFA/SFA index, it is evident that the two groups showed different values (*p* < 0.05), demonstrating the higher unsaturation levels of the SG meat, as reported above. It is also evident that in this index, the effect of monounsaturated fatty acids (MUFAs) was neglected (i.e., possible regulation of plasma LDL concentrations). Indeed, oleic acid (C18:1 n-9), the most representative MUFA in poultry meat, increases the activity of low-density lipoprotein receptors and decreases the cholesterol concentration in serum [38]. In addition, not all SFAs contribute equally to increasing the serum cholesterol concentration.

Even the generalization of the SFA is very explanatory of the negative action of these FAs in increasing the cholesterol concentration in serum by inhibiting the activity of the aforementioned receptors. Indeed, some short chain SFAs are rapidly oxidized by acetyl-CoA in the liver, and they have a very feeble action on LDL receptors; in contrast, C12:0, C14:0, and C16:0 fatty acids show a greater effect on the increase in serum cholesterol. In addition, not all PUFA classes can have the same positive effectiveness toward the prevention of CVD [39]. In general, a value of this index greater than 0.45 is recommended in human diets to prevent CVD and other chronic diseases [40]. Therefore, it is possible to state that the poultry meats analyzed here can be considered of high quality on the basis of this index (especially that produced by SG chickens). In contrast, the same thing cannot be said for the quality of the indexes, which appears outdated due to its scarce specificity toward the single fatty acids and classes. Even the discriminatory capacity, although significant, is low considering the great diversity of the two chicken strains.

Concerning the n-6/n-3 ratio, it should be emphasized that, with an increased specificity of the index, the two experimental groups showed much more evident differences (4.89 vs. 13.04, respectively, for SG and FG birds; *p* < 0.0001). Simoupolus [1] suggests that the human diet in Western countries is unbalanced in terms of the n-6/n-3 ratio (15–16/1 vs. 4/1 recommended), basically because Western diets are deficient in n-3 fatty acids, contrary to what was found in the diets of our ancestors [41]. This situation could promote the pathogenesis of many diseases, including CVD, cancer, and inflammatory and autoimmune diseases, while increased levels of n-3 PUFAs can exert suppressive effects. In the prevention of cardiovascular diseases, a 4/1 ratio was associated with a 70% decrease in total mortality [39]. A ratio of 2.5/1 reduced rectal cell proliferation in colorectal cancer patients, while a ratio of 4/1 with the same amount of n-3 PUFA had no effect [42]. This is consistent with the fact that chronic diseases are multigenic and multifactorial.

Thus, food with a lower n-6/n-3 ratio is more desirable for reducing the risk of many chronic diseases of high prevalence in Western societies. This index shows that SG meat has an equilibrated ratio of PUFAs, whereas FG meat has a much higher ratio. This index is more specific than PUFA/SFA, although it still has some gaps that does not make it suitable for food from animal origins. From a nutritional point of view, the exact content of the various classes of lipids could not completely explain the nutritional value of chicken meat. Even if SG presents a useful lipid profile with an n-6/n-3 ratio of 4.8, closer to the abovementioned desirable 4, the total content of lipids and PUFAs is approximately six times less than that of FG (0.25 vs. 1.45 g/100 g, respectively). These considerations further corroborate the need for developing indexes more specifically dedicated to nutritional quality.

Following this line of evaluation and analyzing the linoleic acid/α-linolenic acid index, it can be seen how strong the effect of the chicken breed is. This index highlights that SG meat is even more interesting, always remaining in a strictly qualitative context. It is common knowledge that the essential PUFAs (LA and ALA) have a different effect on human health, and for this reason, the suggested intake is not equal. LA and ALA compete for the same metabolic pathway in elongase and desaturase reactions to generate active LC-PUFAs. However, the n-3 pathway has a higher metabolic energy expenditure than the n-6 pathway; thus, the latter is the preferential route in the mammalian body, with few exceptions [43,44]. Furthermore, the LA/ALA indexes also showed a first picture of the conversion efficiency of ALA and LA into their longer chain homologs. Harnack et al. [45] suggested that a dietary ALA/LA ratio of 1:1 would lead to the highest formation of n-3 LC-PUFAs, given that the conversion of precursors depends on the n-6/n-3 ratio of the diet. In the above context, even the sum of EPA and DHA (%) gives higher values for SG meat, richer in PUFA, as also demonstrated by the UI and by the most recent references [11,46,47].

The UI represents the relationship between the FA profile and its susceptibility to oxidation, providing useful information on the shelf life of meat. The index highlights the relationship between antioxidant protections and PUFA content and the development of undesirable effects of oxidative stress associated with the formation of lipid peroxides. These processes in turn have been suggested to contribute to the processes of aging and many diseases, such as atherosclerosis [40,48,49,50]. In the present study, meat from FG chickens showed a lower (*p* < 0.0001) value of UI with respect to that of SG, indicating a lower risk of fatty acid autooxidation, but also a lower healthy value of fat obtained from the meat of these lines.

### 3.4. Nutritional Indexes

As already stated, the AI index points out the relationship between the main saturated fatty acids and the main classes of unsaturated fatty acids, considering the former as proatherogenic and as promoters of the activation of immunological cells; thus, they adhere to the vessel wall, whereas the others are antiatherogenic (inhibiting the aggregation of plaque and diminishing the levels of esterified fatty acids, cholesterol and phospholipids, thereby preventing the appearance of micro- and macro-coronary diseases [51]).

The TI indicates the predisposition to form clots in blood vessels. This is defined as the relationship between pro-thrombogenic (saturated) and anti-thrombogenic fatty acids (MUFAs, n-6 PUFAs and n-3 PUFAs). Both AI and TI indicate a potential for stimulating platelet aggregation [52]. Thus, smaller AI and TI values suggest a protective potential for coronary artery health. In terms of human health, an AI and TI in the diet of less than 1.0 and 0.5, respectively, are recommended [53]. However, one critical point of the TI index is the consideration of n-6 PUFAs as anti-thrombogenic agents, whereas in recent years, the relative pro-aggregation properties of n-6 derivatives have been clearly established [38].

Regardless, the obtained values for the meat of the two poultry genotypes can be considered to have high nutritional value, but between them, the SG breast showed the most desirable values. The ratio between hypocholesterolemic and hypercholesterolemic fatty acids indicated the different effects on cholesterol metabolism: higher values are considered more beneficial for human health. In this study, we obtained values for SG and FG chickens of 1.91 and 1.21, respectively (*p* < 0.0001). The health-promoting index, as already reported, represented the inverse of the IA, thus showing inverse values compared with IA (1.81 vs. 1.59, respectively, in SG and FG).

Finally, flesh lipid quality is similar to that of EPA + DHA, calculated as a percentage of total fatty acids. Because it is not affected by lipid content, SG meat showed an almost six-fold higher value than FG meat, underscoring the higher content of n-3 LC-PUFA of the first strain.

### 3.5. Metabolic Indexes

The relevance of these indexes is also connected with their ability to be considered substitutes for expensive laboratory activities [31]. It should be noted that these indexes were developed for identifying the metabolism of animals and have low relevance for discriminating meat quality. Indeed, these indexes are of low nutritional importance because they are not able to discriminate metabolic changes due to dietary effects. All these indexes showed that the SG genotype strongly diverged from the FG genotype. In particular, slow-growing strains had higher levels of elongase, thioesterase and Δ5/Δ6 desaturase, accompanied by a lower Δ9 index, confirming our previous results obtained by comparing native poultry breeds with commercial strains [37].

In fatty acid synthesis, thioesterase is responsible for terminating the reaction and releasing the newly synthesized fatty acid. The ratio of C16:0 to C14:0 could be useful in understanding the selective division of thioesterase on C14-acyl-acyl carrier protein or C16-acyl-acyl carrier protein; in this experiment, the significantly higher thioesterase index observed in SG birds is probably related to less cleavage of C14-acyl-acyl carrier protein. Conversely, the Δ9-desaturase that catalyzes the conversion of C16:0 and C18:0 to C16:1 and C18:1 [54] was lower in SG chickens, suggesting that the lower concentrations of C16:1 in SG chickens (1.32 vs. 16.60 mg/100 g muscle, respectively, for SG and FG) could be attributed to higher Δ9-desaturase activity [55]. An interesting inference comes from the findings of Kouba et al. [56] in pigs, who related the dietary intake of ALA to Δ9-desaturase activity. The same authors observed a negative correlation of this index with the intake of ALA. Our results agree with this assumption, and it could be argued that the higher intake of ALA through forage SG chickens could partly contribute to such Δ9-desaturase lowering. These assumptions are also confirmed by Poureslami et al. [57], who analyzed the interactive effect of diet and age on SFA and MUFA metabolism in poultry, concluding that both factors affected deposition, elongation and Δ9 desaturation activities as well as the oxidation process of fatty acids.

The most evident differences, however, were observed in the Δ5/Δ6-desaturase complex, which represents the most valid tool to verify the capacity of animals to synthesize LC-PUFAs from precursors. The higher Δ5/Δ6-desaturase index (more than double) of the SG genotype demonstrates once again the higher efficiency in LC-PUFA synthesis of the native breeds with respect to commercial hybrids [14,58].

Another interesting finding is related to the competition between n-6 and n-3 fatty acids. A previous investigation [44,58] demonstrated that the n-3 precursor is the preferred substrate in the desaturation and elongation pathway of local breeds with respect to high-performance strains, and that the rate-limiting step in the enzymatic pathways of PUFA biosynthesis could be Δ6-desaturase activity [36,59]. Cherian and Sim [60], investigating the effects of dietary ALA of laying hens on the fatty acid composition of liver microsomes and the activity of Δ6-desaturase in hatched chicks, observed increases in long-chain 20- and 22-carbon fatty acids, which may be attributed to the use of ALA as the preferred substrate over LA. Concordantly, our previous in vitro results revealed that a significant inhibition of Δ-6 desaturase activity occurs with high amounts of ALA in rabbit liver [44].

We can therefore conclude that this category of indexes is efficient in both the discrimination between poultry genotypes characterized by very different lipid metabolism and the replacement of complex and expensive laboratory analyses for the determination of enzymatic activity in microsomes.

The activity index is based on the indirect evaluation of β-oxidation activity in red and white chicken muscles to estimate their energy expenditure. In our case, the two genotypes showed differences (1.97 vs. 1.28, *p* < 0.0001, respectively, for SG and FG), magnified by the extensive rearing system, where the birds were allowed to walk in an outdoor run. The activity index is therefore an attempt to measure the energy expenditure due to the movement developed throughout the entire life of the chicken, with all the qualitative and nutritional consequences that this entails in its meat. This index can only be measured by evaluating the FA profile of the muscle after slaughter, and is surely a valid and objective tool for measuring the adaptability of a genetic line to extensive rearing systems, because it accounts for all the activity exerted during the life of the animal and can be used for an ex post welfare assessment.

### 3.6. Lipid- or Energy Content-Related Indexes

The analysis of all the indexes considered thus far have highlighted some strengths, such as the discriminatory capacity between different poultry genotypes and the effectiveness in replacing complex and expensive analytical practices. However, many gaps have been highlighted, mainly from the nutritional viewpoint. To fill these gaps, our idea was to relate the content of fatty acids with high nutritional value with that of total lipids, in the belief that the two parameters have to be considered together. Establishing precise relationships between these components can provide interesting and definitive information on the nutritional value of a food.

The sum of EPA and DHA (in mg/100 g f.m.; previously discussed as a percentage of the total fatty acids) is very important, as it is well known that the roles of n-3 LC-PUFAs in human metabolism [27] provide information on their absolute presence, which is inextricably linked to the quantities of meat lipids. This index is recognized worldwide, and recommendations for these two fatty acids and their precursors have been made by the Food and Agriculture Organization of the United Nations. In particular, 0.5–0.6% ALA per day is useful to prevent deficiency symptoms, whereas the n-3 LC-PUFA recommendation is approximately 250–2000 mg per day. For adult males and nonpregnant/nonlactating females, 250 mg/day EPA plus DHA is recommended, with insufficient evidence to set a specific minimum intake of either EPA or DHA. For adult pregnant and lactating females, the minimum intake for optimal adult health and fetal and infant development is 300 mg/d EPA + DHA, of which at least 200 mg/d should be DHA [39].

As the last index, we wanted to simulate a portion of 150 g of chicken meat containing approximately 33 g of proteins, which represents a correct amount to cover the protein requirement of a balanced meal (20% of total energy in a 1800 kcal balanced Mediterranean diet). In this context, investigating the lipid contribution of a standard portion of chicken in a balanced Mediterranean diet could allow us to establish a dietary plan with other sources of lipids (i.e., olive oil) to both reach the right amount of lipid-derived energy per meal (approximately 35% of energy expressed in calories) and counterbalance levels of different PUFA classes (n-6/n-3 ratio).

Chicken meat obviously cannot represent the only source of lipids in a balanced diet, but knowing its exact lipid composition and the dietary implications could help in the elaboration of a high-quality dietary plan. This analysis could also allow meat scientists, clinicians, and experts in nutrition to engage in better dialog for improving the lipid content of chicken meat by intervening in farming techniques, nutrition and additives for livestock. Undoubtedly, this manuscript allows nutrition experts to gain in-depth knowledge of lipid quality in terms of health promotion and preservation and to elaborate balanced dietary plans.

With a portion of SG or FG breast meat in a standard meal, we can reach EPA plus DHA intake levels of 5.58 and 4.83 mg, respectively. These quantities are low considering the differences between terrestrial plants and animals with respect to seafood [61], particularly fish, but surely interesting considering the low lipid contents of these meats. Indeed, it should be considered that deskinned breast meat has a very low-fat content: the same portions obtained from other body parts (i.e., drumstick, thigh) have quite the same fatty acid profile, but with approximately 10 times more fat, with up to 20–25% of the daily recommendation. A further increase in n-3 LC-PUFAs of meat could also be attained with dietary supranutritional administration of LNA [56].

The large difference between the two genotypes lies in the fact that in the SG meat, the content depends on the high unsaturation, while in the FG meat, it depends on the high content of lipids (relative to SG, of course), which in any case provides good quantities of EPA and DHA.

The amount of 150 grams of chicken contains approximately 100 mg of n-6 in SG meat vs. 500 mg in FG meat and 15 mg of n-3 in SG vs. 40 in FG. Considering 1 g of n-3 as an adequate dose for a meal, we see that the percentages of both SG and FG are very low, demonstrating that chicken breast meat cannot represent a primary source of lipids, but at the same time, these data give us information on how to build a balanced diet by using meat containing good lipids that at the same time bring the n-6/n-3 ratio more toward 4/1 and provide the right amount of lipids. At this point of the discussion, a relevant question arose: are the high quantities of n-3 LC-PUFA contained in foods rich in lipids advisable in a healthy human diet? Is it, perhaps, necessary to deepen the relationship between these components?

INQ, which relates the quantities of EPA and DHA to the energy content of the food (weighted for the daily requirements of these two fatty acids), offers more precise nutritional information, emphasizing the goodness of the SG meat (high PUFA and low-fat content), but as previously mentioned, a simultaneous relationship between fat and the fatty acid profile is needed. In particular, the QuantiN-3 index relates the polyunsaturated fatty acids of the n-3 series expressed in mg/100 g meat with the quantity of fat (g/100 g). Because the concentration of fatty acids with respect to the total lipids varies from 0.83 to 0.91 on the basis of animal species [56], we also considered going into more detail with regard to the total amount of fat added.

Based on the critical analysis of the indexes previously reported, we constructed two indexes (healthy fatty indexes 1 and 2). The concept of HFI1, which then evolved into HFI2, is to give different weights to the various categories of fatty acids, relying on their healthy properties. In particular, in the first-mentioned index, all fatty acids with more or less pronounced positive action on human health are reported in the numerator. All classes were multiplied by empirical constants partly derived from Ulbricht and Southgate [7], two for MUFAs, four for n-6, and eight for n-3, to consider the nutritional and health implications of the various classes.

As in the Quanti n-3 index, the denominator shows the total lipids. Fatty meat lowers the index and the relative nutritional quality; however, not all lipids have the same nutritional effect, which must be represented in the index. Hence, in building a new index (HFI2), nothing changes in the numerator, but the lipids in the denominator are given different weights, obviously in the opposite manner to that described above. SFAs are considered fully, whereas MUFAs, n-6 PUFAs and n-3 PUFAs are reduced by half, a quarter and an eighth, respectively, using the math fraction of the constants in the numerator to consider their less negative effect. Furthermore, the n-3/n-6 ratio (with the ratio in a positive sense, i.e., reversed ratio) was introduced to consider the healthy importance of their balance.

In our opinion, this careful categorization of the different classes of fatty acids can allow a comprehensive lipid indexing of food, taking into consideration their quality and nutritional characteristics, with an evaluation of the quantities of lipids that carry these bioactive compounds. In other words, is it better to eat fatty meat that still brings many n-3 PUFAs even if not very rich in percentages, or is a lean meat with a high percentage of unsaturation preferred?

From the analysis of the HFI2 in Table 3, there is no doubt, as the lean meat of SG chickens showed significantly (*p* < 0.0001) higher values than that of FG chickens (3.22 vs. 2.80, respectively); however, increasing the quantity of lipids should not be demonized if the meat presents healthy FA classes, especially in a monogastric species such as chicken. In our case, hypothetically bringing the levels of n-3 in FG meat to the levels observed in SG meat, the HFI2 index would rise to 3.22, a value even higher than that of the very lean SG meat.

## 4. Conclusions

This study clearly demonstrates how complex it is to index fatty acids in order to assess the nutritional or health potential of chicken meat. Our conclusion is related to the concept that an overall and final evaluation must consider the many factors that regulate the nutritional properties of food. In particular, the level of fats, the FA profiles and the relationships between them is of fundamental importance for the design and adoption of an index. We believe that Healthy Fatty Index 2 collects all this information. It is tentative and will certainly require adjustment in light of further evidence, especially in the assessment of the constant values.

Further investigations are necessary to better define the weights of the different classes of fatty acids (or of the single fatty acid), the discriminatory capacity of this index (within different foods) and its parameterization with a standard dose of meat for consumption. Moreover, when Healthy Fatty Index 2 will be validated, it is our intention to broaden our horizon to other meats and foods (both of animal and vegetable origin) in order to create a new-generation nutritional quality database.

## Figures and Tables

**Table 1:** Ingredient composition and calculated analysis of diets.

	Starter	Finisher
Ingredients		
Maize	52.0	46.0
Full-fat soybean	30.5	12.5
Wheat	-	20.0
Soybean meal	9.00	14.0
Alfalfa meal	2.80	2.80
Corn gluten feed	3.00	2.00
Vitamin-mineral premix ^(1)^	1.00	1.00
Dicalcium phosphate	1.00	1.00
Sodium bicarbonate	0.50	0.50
NaCl	0.20	0.20
Chemical composition		
Dry matter	90.9	90.8
Crude protein (%)	22.3	18.0
Ether extract (%)	7.95	4.98
Crude fiber (%)	4.67	4.01
Ash (%)	5.76	5.59
NDF—Neutral Detergent Fiber	10.7	10.1
ADF—Acid Detergent Fiber	5.58	5.06
Cellulose (%)	4.22	3.56
ADL—Acid Detergent Liquid	1.03	1.11
Hemicellulose (%)	5.16	5.05
Metabolizable Energy (Mj/kg DM)	12.5	12.9

^(1)^ Amounts per kg: Vit. A 11.000 IU; Vit. D3 2.000 IU; Vit. B1 2.5 mg; Vit. B2 4 mg; Vit. B6 1.25 mg; Vit. B12 0.01 mg; α-tocopheryl acetate 30 mg; biotin 0.06 mg; Vit. K 2.5 mg; niacin 15 mg; folic acid 0.30 mg; pantothenic acid 10 mg; choline chloride 600 mg; Mn 60 mg; Fe 50 mg; Zn 15 mg; I 0.5 mg; Co 0.5 mg.

**Table 2:** Summary of the studied indexes and their significance regarding health effects (D = direct; I = indirect).

Sub- Categories	Indexes	Unit	Sign.	References
Qualitative	PUFA/SFA n-6/n-3 ratio LA/ALA EPA + DHA% Unsaturation Index (UI)	%	D	Many Authors
		%	I	Simopoulus, 2008 [1];
		%	I	Undurti, 2006 [2];
		%	D	Holub, 2009 [3], Crupi and Cuzzocreas, 2022 [4];
		%	I	Shahidi and Zhong, 2010 [5].
Nutritional	Nutrition Value Index Index of Atherogenicity (IA) Index of Thrombogenicity (IT) Hypocholesterolemic/Hypercholesterolemic (HH) Health-Promoting Index (HPI) Fish Lipid Quality/Flesh Lipid Quality (FLQ)	%	D	Chen et al., 2016 [6];
		%	I	Ulbricht and Southgate, 1991 [7];
		%	I	Ulbricht and Southgate, 1991 [7];
		%	D	Santos-Silva et al., 2002 [8];
		%	D	Chen et al., 2004 [9];
		%	D	Xie et al., 2022 [10].
Metabolic	Elongase Thioesterase Δ 9-desaturase (18.0) Δ 9-desaturase (16.0 + 18.0) Δ5-desaturase + Δ6-desaturase Activity index	%	-	Zhang et al., 2007 [12]];
		%	-	Zhang et al., 2007 [12];
		%	-	Vessby et al., 2002 [13];
		%	-	Vessby et al., 2002 [13];
		%	-	Sirri et al., 2011 [14];
		%	-	Failla et al., 2021 [19].
Lipid- or energy-related content indexes	EPA + DHA quantity Index of nutritional quality QuantiN-3 index Healthy fatty index 1 Healthy fatty index 2	mg/100 g	D	Godbe, 1994 [20];
		mg/100 g	D	Sorenson et al., 1976 [21];
		mg/100 g	D	Present paper;
		mg/100 g	D	Present paper;
		mg/100 g	D	Present paper.

PUFA: polyunsaturated fatty acid; SFA: saturated fatty acid; LA: linoleic acid; ALA: α-linolenic acid; EPA: eicosapentaenoic acid; DHA: docosahexaenoic acid.

**Table 3:** Lipid contents (g/100 g meat) and fatty acid compositions (g/100 g fatty acids and mg/100 g meat) of breast meat from different genotypes.

	SG	FG	SEM	SG	FG	SEM
Lipids (g/100 g meat)	0.25 B	1.45 A	0.13			
	% of total FA			mg/100 g meat		
C12:0	0.20 B	0.62 A	0.09	0.43 B	7.41 A	1.03
C14:0	0.71 B	1.04 A	0.21	1.54 B	12.42 A	2.21
C16:0	27.98 b	30.01 a	2.03	60.53 B	358.45 A	125.14
C18:0	12.02 a	11.00 b	1.61	30.33 B	131.39 A	20.14
Others	2.49	2.07	0.20	7.55 B	24.72 A	3.56
Total SFA	43.40 a	44.74 b	2.11	100.38 B	534.39 A	156.48
C14:1n-6	0.15	0.11	0.04	0.32 B	1.31 A	0.24
C16:1n-7	0.61 A	1.39 B	0.19	1.32 B	16.60 A	3.14
C18:1n-9	19.50 A	23.04 B	2.31	40.02 B	275.20 A	10.41
Others	0.26	0.17	0.91	0.56 a	2.03 b	0.48
Total MUFA	20.52 A	23.94 B	1.75	42.23 B	293.95 A	23.47
C18:2n-6	18.09 B	20.70 A	2.18	39.14 B	247.25 A	25.36
C20:2n-6	0.68 a	0.20 b	0.16	1.47 b	2.39 a	0.23
C20:3n-6	0.17	0.16	0.03	0.37 B	1.91 A	0.47
C20:4n-6	10.60 a	8.03 b	1.36	22.93 B	95.91 A	15.98
Total n-6	29.54	29.09	2.47	63.91 B	347.46 A	36.98
C18:3n-3	2.03 a	0.98 b	0.74	2.23 B	11.71 A	4.12
C20:3n-3	0.04	0.03	0.01	0.09 B	0.36 A	0.13
C20:5n-3	0.68 A	0.09 B	0.27	0.39 B	1.07 A	0.32
C21:5n-3	0.87 A	0.03 B	0.47	1.88 A	0.36 B	0.24
C22:5n-3	1.37 A	0.92 B	0.25	2.96 B	10.99 A	1.79
C22:6n-3	1.05 A	0.18 B	0.29	2.26	2.15	0.14
Total n-3	4.54 A	2.23 B	1.75	9.81 B	26.64 A	3.49
Total PUFA	35.58 A	31.32 B	3.04	73.72 B	374.10 A	98.85

SG: Slow-growing; FG: Fast-growing; SEM: standard error mean; SFA: saturated fatty acids; MUFA: monounsaturated fatty acids; PUFA: polyunsaturated fatty acids; Number: 20 samples per group; A,B: *p* < 0.0001; a,b: *p* < 0.05.

**Table 4:** Indexes of breast muscle of different chicken genotypes.

Indexes	SG	FG	SEM
Qualitative			
Polyunsaturated/Saturated Fatty Acids	0.81 a	0.70 b	0.06
n-6/n-3 Fatty Acids Ratio	4.89 B	13.04 A	1.89
Linoleic Acid/α-Linolenic Acid	8.91 B	21.12 A	2.15
Sum of EPA and DHA	1.73 A	0.27 B	0.23
Unsaturation Index	128.05 A	107.75 B	5.36
Nutritional			
Nutritional Value Index	1.11	1.14	0.14
Index of Atherogenicity	0.55 b	0.63 a	0.07
Index of Thrombogenicity	0.94 b	1.24 a	0.10
Hypocholesterolemic/Hypercholesterolemic	1.91 A	1.21 B	0.12
Health-Promoting Index	1.81 a	1.59 b	0.17
Fish Lipid Quality/Flesh Lipid Quality	1.73 A	0.27 B	0.12
Metabolic			
Elongase Index	0.47 a	b	0.09
Thioesterase Index	42.7 A	30.9 B	4.23
Δ9-Desaturase (18)	58.0 B	68.7 A	3.65
Δ9-Desaturase (16 + 18)	31.40 b	37.51 a	6.32
Δ5/Δ6-Desaturase	52.48 A	23.55 B	4.92
Activity index	1.97 A	1.28 B	0.12
Lipid- or energy-related content indexes			
Sum of EPA and DHA (mg/100 g meat)	3.72 a	3.22 b	0.15
Index of Nutritional Quality	9.42 A	7.29 B	0.74
Quanti n-3 Index	53.23 A	18.37 B	4.05
Healthy Fatty Index 1	1.80 a	1.49 b	0.08
Healthy Fatty Index 2	3.22 A	2.80 B	0.12

Calculated on 20 samples per group; A,B on the same line: *p* < 0.0001; a,b: *p* < 0.05.

## Data Availability

The data are available upon justified request.
